# Metabolomic Approach for Characterization of Polyphenolic Compounds in *Laminaria japonica*, *Undaria pinnatifida*, *Sargassum fusiforme* and *Ascophyllum nodosum*

**DOI:** 10.3390/foods10010192

**Published:** 2021-01-19

**Authors:** Ping Shen, Yue Gu, Chunxu Zhang, Chenghang Sun, Lei Qin, Chenxu Yu, Hang Qi

**Affiliations:** 1 School of Food Science and Technology, Dalian Polytechnic University, Dalian 116034, China; shenping_dpu@126.com (P.S.); gu990713@163.com (Y.G.); qinlei@dlpu.edu.cn (L.Q.); 2 School of Mechanical Engineering and Automation, Dalian Polytechnic University, Dalian 116034, China; zcx747189502@163.com; 3 Department of Biochemical Engineering, Chaoyang Teachers College, Chaoyang 122000, China; Sunchenghang2020@163.com; 4 Department of Agricultural and Biosystems Engineering, Iowa State University, Ames, IA 50011, USA; chenxuyu@iastate.edu

**Keywords:** brown macroalgae, polyphenolics, phlorotannins, antioxidant activity, Q-Exactive HF-X, multivariate data analysis

## Abstract

Profiling of polyphenolics in four types of brown macroalgae, namely *Laminaria japonica* (*L. japonica*), *Undaria pinnatifida* (*U. pinnatifida*), *Sargassum fusiforme* (*S. fusiforme*), and *Ascophyllum nodosum* (*A. nodosum*), and their effect on oxidation resistance were investigated for the first time. Polyphenolic extracts from marine brown macroalgae were shown to effectively remove oxidants from cells and cellular systems. *A. nodosum* showed the highest antioxidant activity among evaluated brown macroalgae, showing a better scavenging effect on 2,2-diphenyl-1-picrylhydrazyl (DPPH) free radical and alleviating oxidative damage caused by hydrogen peroxide to human keratinocytes (HaCaT) cells. Through Q-Exactive HF-X mass spectrometry analysis, 12 polyphenolic compounds were preliminarily identified, including phlorotannins, phenolic acids, and flavonoids. Significant differences in content and variety of polyphenolics were found in evaluated brown macroalgae, which could be related to differences in antioxidant activity in vivo and in vitro. Moreover, the antioxidant activity might be related to the total phenolic content and the types of polyphenolics, especially phlorotannins. The findings presented in this study indicate that *A. nodosum* could be used as an important substitute for functional ingredients in foods and pharmaceutical preparations, as well as a raw material for phlorotannins research.

## 1. Introduction

In order to replace the previously used synthetic ingredients and potentially harmful ingredients, the industry is now focusing its efforts and resources on the discovery and application of natural biologically active compounds [1,2]. In the last ten years, the marine ecosystem has been attracting the attention of researchers worldwide as it is a rich source of organisms possessing or producing substances with high biological activity. In particular, marine macroalgae (seaweeds) are a type of heterogeneous photosynthetic organisms different from land plants [3], having great potential industrial applications. In 2016, the global production of commercial aquatic plants (including macroalgae) reached 31.2 million tons, of which macroalgae aquaculture accounted for 96.5% [4]. Macroalgae are often exposed to unfavorable environmental conditions that can lead to deleterious effects that are often not readily visible, hence a wide variety of metabolites (pigments, polysaccharides, polyphenolics, and so on) are produced by macroalgae to neutralize such effects [5]. Macroalgae are mainly divided into three orders, namely *Laminariales*, *Fucales,* and *Dictyotales*. In particular, the phytochemical composition of *Laminaria*, *Sargassum*, *Ascophyllum*, *Ecklonia*, *Undaria*, *Himanthalia,* and *Dictyota* have been widely studied [6].

Polyphenolics are derived from the secondary metabolism of plants, whose structure is formed by an aromatic benzene ring with one or more hydroxyl bonded directly to the aromatic carbon, which includes all functional derivatives [7]. Among phytochemicals, polyphenolics can counteract dangerous reactive oxygen species (ROS) due to their high redox potential, thus acting as reducing agents, hydrogen donors, singlet oxygen quenchers, and metal chelators [8]. The polyphenolic compounds commonly found in terrestrial plants are gallic and ellagic acid. However, polyphenolics in brown macroalgae vary from simple phenols (such as phenolic acids with a single aromatic structure) to highly complex compounds called phlorotannins, which are synthesized by polymerization of phloroglucinol (1,3,5-trihydroxybenzene) units [9]. The biosynthetic pathway for phlorotannins biosynthesis is still ill-defined, but it has been suggested that phloroglucinol is formed by the acetate-malonate (or polyketide) pathway [10]. The production of phlorotannins is limited to brown macroalgae which are known to occur in soluble (in the cytoplasm or within cell organelles) or in cell wall-bound forms, similar to other tannins [11]. Phlorotannins play a variety of roles in brown macroalgae, both at the cellular and organismal level, from early developmental stages to adult plants, especially in alleviating the oxidative damage caused by high oxygen concentration in the environment [12]. Phlorotannins possess a heterogeneous and high molecular weight group of compounds, in the dry algae in an amount up to 20% [13]. Phlorotannins can be divided into five major subclasses according to the nature of structural linkages between phloroglucinol units and the number and distribution of hydroxyl groups: fucols, phlorethols, fucophlorethols, eckols, and fuhalols [14]. Phlorotannins have been increasingly investigated for their wide array of bioactivities [15], including antioxidant [16], anti-inflammatory [17], anticancer [18], and antidiabetic [19]; thus, macroalgae-derived products rich in bioactive components such as phlorotannins show promising commercial potential in the food and pharmaceutical sectors [20].

Current knowledge on polyphenolics in brown macroalgae derives from research performed on crude extracts based on colorimetric methods (Folin-Ciocalteu assay). Nonetheless, it is limited by their chemical characterization, mainly due to the complexity and reactivity of structure [21]. Hence, more in-depth studies are needed for the adequate identification and characterization of polyphenols in brown macroalgae. The availability of advanced chromatography and mass spectrometry techniques allows the possibility to tentatively identify polyphenols [22] since the chemical characterization is crucial to understand both the ecological and commercial relevance of polyphenols. Moreover, it has been reported that phlorotannins bioactivities are dependent on the molecular weight of the compound: a higher degree of polymerization of the compound has been associated with enhanced activity, as a higher number of phenolic rings is associated with a greater number of hydroxyl groups, which is the reducing component [23,24]. Moreover, the arrangement of such hydroxyl groups around the phlorotannin ring also impacts reactivity. Marine macroalgae can by adjusting the content and composition of intracellular metabolites, in time and space to adapt to the surrounding environment. In addition, it is also largely affected by species genotype [25]. However, the association between species and changes in the metabolic profile and biological activity of polyphenols has not been yet elucidated.

Considering the potential use of brown macroalgae as a natural source of polyphenolic compounds with antioxidant activity, determining which species of brown algae are abundant in polyphenolics is a crucial step. Therefore, the present study aimed to evaluate antioxidant activity in four types of brown macroalgae, namely *Laminaria japonica*, *Undaria pinnatifida*, *Sargassum fusiforme,* and *Ascophyllum nodosum*; identify polyphenolic compounds using mass spectrometry and perform multivariate data analysis; and determine the association between polyphenolic components and antioxidant activity. The above research can provide a strong basis for the application of polyphenolics in brown macroalgae.

## 2. Materials and Methods 

### 2.1. Chemicals and Reagents

Folin Ciocalteu reagent and fetal bovine serum (FBS) were purchased from Shenggong Bioengineering Co., Ltd. (Shanghai, China). 2,2-diphenyl-1-picrylhydrazyl (DPPH) was purchased from Sigma-Aldrich (St. Louis, MI, USA). Dulbecco’s Modified Eagle Medium (DMEM) (Gibco, Waltham, MA, USA) and Penicillin-streptomycin (HyClone™) were purchased from Beijing Baoxidi Co., Ltd. (Beijing, China). 3-(4,5-dimethylthiazol-2-yl)-2,5-diphenyltetrazolium bromide (MTT) and dimethylsulfoxide (DMSO) were purchased from Soleibao Technology Co., Ltd. (Beijing, China). Methanol and acetonitrile were HPLC grade, and other reagents were analytical grade produced by local companies in China.

### 2.2. Brown Macroalgae Samples

*L. japonica* and *U. pinnatifida* produced from the Yellow Sea in Liaoning, China, as well as *S. fusiforme* produced from the East Sea in Zhejiang, China, were purchased from Dalian local supermarkets (Liaoning, China). *A. nodosum* produced in Chile was supplied by Qingdao Mingyue Algae Group Co., Ltd. in Shandong Province, China. During transportation, 4 kinds of brown macroalgae materials were dried and sealed. After removal of impurities, macroalgae samples were dried at 50 °C and ground to a powder (particle size < 0.9 mm). Powdered macroalgae samples were vacuum-packed and stored below −30 °C until further experiments.

### 2.3. Sample Preparation

Polyphenolic compounds were extracted following the method described by Ummat et al. [26] with minor modifications. Briefly, the powdered sample was mixed with 50% ethanol (1:15, *w/v*) and, after an ultrasonic treatment for 30 min, the mixture was incubated in a shaking water bath at 50 °C under 120 rpm for 8 h. Supernatant was then filtered through Whatman #1 filter paper (Whatman International Limited, Maidstone, UK) and ethanol was evaporated (70 rpm, 50 °C, 0.1 MPa) in a rotary evaporator (SY-2000, Shanghai Yarong Biochemical Instrument Factory, China). The remaining aqueous mixture was loaded onto a Sep-Pak C18 Plus Short Cartridge (500 mg sorbent per cartridge, 3 mL column volume, 50/pkg) (Agela, Beijing, China), which was previously conditioned with methanol followed by water. Polyphenolic compounds were eluted with methanol and the solvent was evaporated under reduced pressure until completely dry. Dry polyphenolic extracts were vacuum sealed and stored at −80 °C until analyses were performed.

### 2.4. Determination of Total Phenolic Content

Total phenolic content was determined as gallic acid equivalents (GAE) using the Folin–Ciocalteu reagent [27]. The macroalgae phenolic extract (50 µL) was diluted with deionized water (750 µL), Folin-Ciocalteu phenol reagent (50 µL) was added, and contents were mixed thoroughly. After 1 min, 150 µL of 20% sodium carbonate solution was added, followed by thorough homogenization. After incubation for 1 h at 37 °C, absorbance was measured at 760 nm using a Tecan infinite 200 Microplate Reader (Tecan Trading AG, Switzerland). The measured value was compared to a standard curve prepared with a gallic acid solution. Total phenolic content was expressed as GAE per gram of dried extract.

### 2.5. Profiling of Polyphenolic Compounds by Mass Spectrometry

Analyses were performed in a Q-Exactive HF-X Hybrid Quadrupole-Orbitrap (Q-Exactive HF-X) mass spectrometer with electrospray ionization (ESI) using a Dionex UltiMate 3000 system (Dionex Softron GmbH, Germany) with an Acquity UPLC BEH HILIC column (2.1 × 150 mm, particle size 1.7 μm; Waters Corporation, Milford, MA, USA). Run conditions were: 65 °C column temperature; 0.4 mL/min final flow rate; 1 μL injection volume; 10 °C chamber temperature. Mobile phase A was 0.1% formic acid in water. Mobile phase B was acetonitrile: water (95:5, *v/v*) containing 0.1% formic acid. Polyphenols were separated by the following gradient elution: 0–2 min 100% B; 2–7.7 min 100–70% B; 7.7–9.5 min 70–40% B; 9.50–10.250 min 40–30% B; 12.250–12.750 min 30–100% B; 12.750–17.000 min 100% B. Spectra acquisition was performed in positive ionization modes, and spectra were acquired over a mass range of m/z 60–900. In both modes, the sheath gas flow rate was 60%, the aux gas flow rate was 20%, the sweep gas flow rate was 1%. Spray voltage was at 3.6 kV, capillary temperature was 380 °C, aux gas heater temperature was 370 °C. All acquisitions were performed using D3–1-methylnicotinamide (m/z 141.0976) for lock mass calibration. Four analytical replicates from each sample were analyzed in Q-Exactive HF-X. In order to relatively quantify the polyphenolic compounds of brown macroalgae, the final concentration of polyphenolic extracts of four kinds of brown macroalgae was 2 mg/mL, and the dissolving solvent was methanol.

Compounds were identified according to their m/z ratio in the ESI mass spectrometer. Identified compounds were compared against analytical standards and available databases based on their neutral mass isotope distribution, retention time, and MS/MS fragments using a customized database of polyphenolics on PubChem (https://pubchem.ncbi.nlm.nih.gov/), online metabolite databases METLIN (http://metlin.scripps.edu/) and MassBank (http://www.massbank.jp), and previously published data.

### 2.6. DPPH Radical Scavenging Ability by Electron Spin Resonance

Electron spin resonance (ESR), also known as electron magnetic resonance (EMR) or electron paramagnetic resonance (EPR), is the most effective and direct method to measure free radicals (such as DPPH radical) [28]. Detection of free radicals was performed by ESR spectroscopy (A200, Bruker, Karisruhe, Germany) according to Qi et al. [29] with a few modifications. Sample concentration for the determination of DPPH free radical scavenging ability was set at 40 μg/mL; ESR scanning conditions were: 3477.5 G central magnetic field; 100 G scanning width; 5.21 mW microwave power; 40 ms conversion time; and 81.92 ms time constant. Each sample was scanned at least three times. Sample concentration for the determination of hydroxyl radical scavenging ability was 20 μg/mL; ESR scanning conditions were: 3470 G central magnetic field, 80 G scanning width, 6.08 mW microwave power, 360 ms conversion time, and 5242.88 ms time constant.

DPPH radical scavenging ability was calculated as follows (1):

DPPH radical scavenging ability (%) = A_0_ − A/A_0_ × 100 (1)

where A_0_ is the absorbance of the control and A is the absorbance of samples/standards.

### 2.7. Cell Culture

Human epidermal keratinocyte (HaCaT) cells were obtained from Tong-Pai Bio-Technology Co., Ltd. (Shanghai, China). Cells were kept in DMEM high glucose and enhanced with 10% FBS and 1% penicillin-streptomycin in a humidified incubator under 5% CO_2_ at 37 °C. Samples were prepared by dissolution in dimethyl sulfoxide (DMSO). Stock solutions were diluted to desired concentrations using complete DMEM.

### 2.8. Cell Viability

The MTT assay was used to detect the effect of polyphenol extracts from brown macroalgae on the oxidative damage induced by H_2_O_2_ in HaCaT cells. Cells (1.5 × 10^5^ cells/well) were plated on a 96-well plate and cultured for 24 h. After that, cells were treated with H_2_O_2_ (1 mM) for an initial 1 h and then with the polyphenolic extract (5 μg/mL) for 23 h. MTT stock solution (5 mg/mL) was added to the wells, and cells were incubated for 4 h to yield formazan crystals. After the dissolution of crystals in DMSO, absorbance was measured at 570 nm using a multiwell scanning spectrophotometer. Control (untreated cells) was considered as 100% viability and resulting cell viability was calculated as (2):

Cell viability(%) = A_sample_/A_control_×100 (2)

where A_sample_ is the absorbance of the sample and A_control_ is the absorbance of control.

### 2.9. Statistical Analysis

All experiments were repeated at least three times. Differences between samples for each analyzed polyphenolic compounds were tested with one-way analysis of variance (ANOVA) in SPSS 20.0 (SPSS Inc., Chicago, IL, USA). *p*-value < 0.05 was considered statistically significant. Principal component analysis (PCA), partial least squares discriminant analysis (PLS-DA), and heatmap clustering analysis were performed in MetaboAnalyst 4.0.

## 3. Results and Discussion

### 3.1. Evaluation of Total Phenolic Content and Antioxidant Activity 

**Figure 1:**
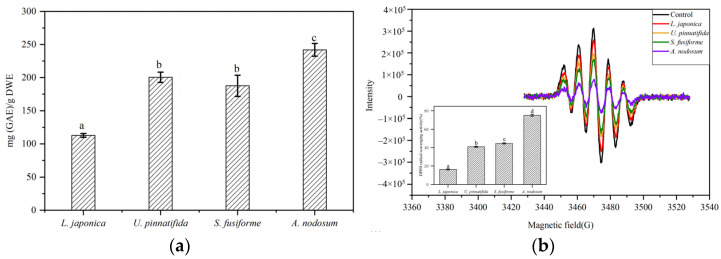

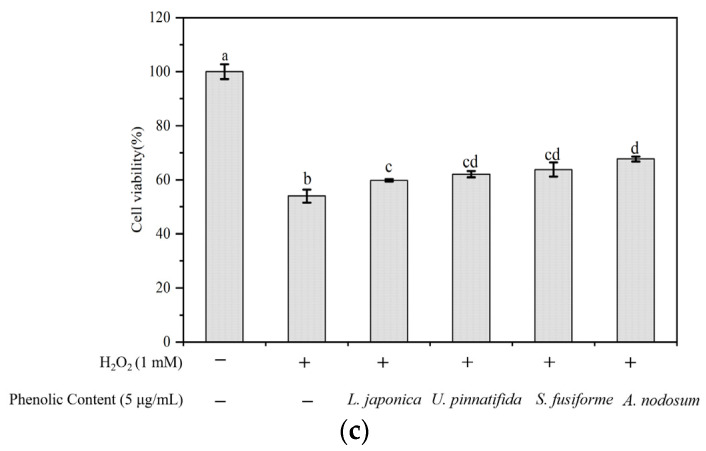
Evaluation of total phenolic content and antioxidant activity in four types of brown macroalgae in vitro and in vivo. (**a**) Total phenolic content (mg GAE/g dry weight extract) (**b**) 2,2-diphenyl-1-picrylhydrazyl (DPPH) radical scavenging activity (**c**) Cell viability after challenge with H_2_O_2_. Data are presented as mean ± standard deviation. Different letters in the same row indicate significant differences as assessed by Duncan’s multiple-range test following one-way ANOVA (*p* < 0.05).

Total phenolic content in four brown macroalgae was determined by the Folin-Ciocalteu colorimetric method. Total phenolic content in the evaluated macroalgae varied significantly, ranging from 112.69 ± 2.85 to 241.85 ± 9.62 (mg GAE/g dried extract) (Figure 1a). Total phenolic content was highest in *A. nodosum* (241.85 ± 9.62) and lowest in *L. japonica* (112.69 ± 2.85), which are in line with similar studies. Kirke et al. [30] studied changes in total phenolic content in *A. nodosum* during different seasons, which ranged from 50–250 mg GAE/g dried extract. Similar phenolic content was reported in *Ecklonia kurome* (97 mg PGE/g) [31], *Ecklonia bicyclis* (192.8 mg GAE/g) [32], and *Laminaria ochroleuca* (173.65 mg GAE/g) [33].

DPPH radical scavenging ability of polyphenolics extracted from evaluated brown macroalgae was analyzed by ESR with spin trapping. The polyphenolics extracted from *A. nodosum* (75.03 ± 1.07%) showed significantly strong (*p* < 0.05) scavenging activity when compared to *S. fusiforme* (44.62 ± 0.62%), *U. pinnatifida* (40.97 ± 0.59%) and *L. japonica* (16.30 ± 0.65%) (Figure 1b).

Previous studies have found that polyphenolics in brown macroalgae can protect cells against oxidative damage induced by H_2_O_2_. Of notice, polyphenolics in marine macroalgae were shown to effectively reduce the death of Chinese hamster lung fibroblasts induced by H_2_O_2_, as well as inhibit radiation-induced cell damage and eliminate the production of ROS [34]. In the present study, the protective effects of polyphenolic extracts from brown macroalgae against oxidative damage induced by H_2_O_2_ in HaCaT cells were evaluated and cell viability was measured by the MTT method. H_2_O_2_-stimulated cells without polyphenolic extracts showed 54.01 ± 2.45% cell survival rate, while rates of survival were increased when cells were pre-treated with a non-toxic concentration (5.00 µg/mL) of polyphenols extracted from *L. japonica* (59.84 ± 0.50%), *U. pinnatifida* (62.09 ± 1.15%), *S. fusiforme* (63.82 ± 2.60%) and *A. nodosum* (67.67 ± 0.96%) (Figure 1c).

Interestingly, total phenolic contents differed significantly among the evaluated types of brown macroalgae; however, changes in antioxidant activity were not affected by total phenolic content. For example, the total phenolic content of *U. pinnatifida* was higher than in *S. fusiforme*; this might be explained by the fact that the Folin-Ciocalteu method not only measures the reducing capacity of polyphenolic compounds but also that of other reducing substances such as ascorbic acid, aromatic amines and sugars [35]. In addition, the degree of antioxidant activity observed in the evaluated macroalgae might not only be due to the higher polyphenol content but also due to the geometric arrangement of the polyphenol structure and the location of free radicals [24].

### 3.2. Identification of Polyphenolic Compounds by Q-Exactive HF-X Mass Spectrometry

**Figure 2:**
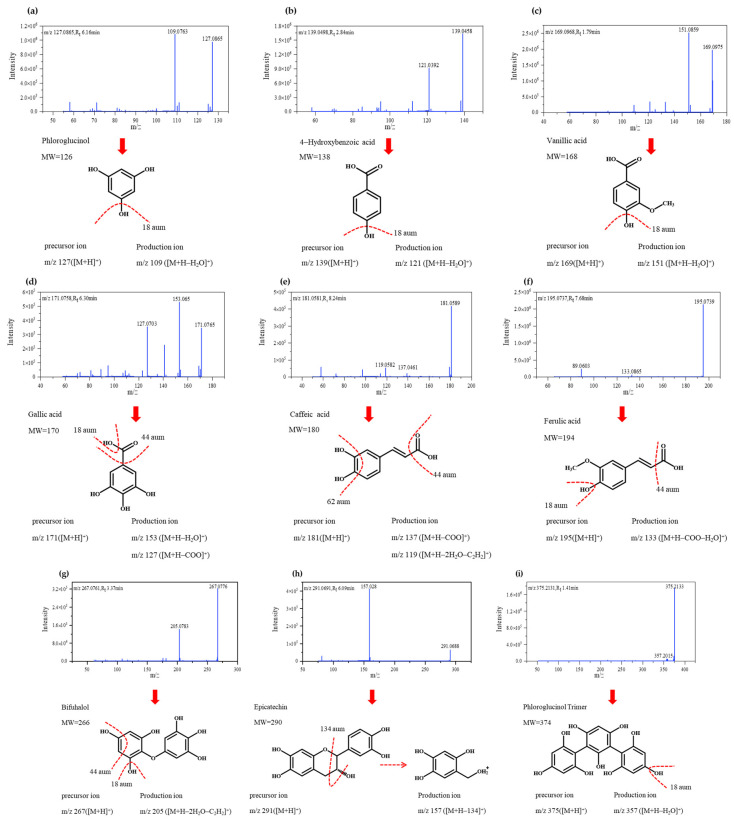

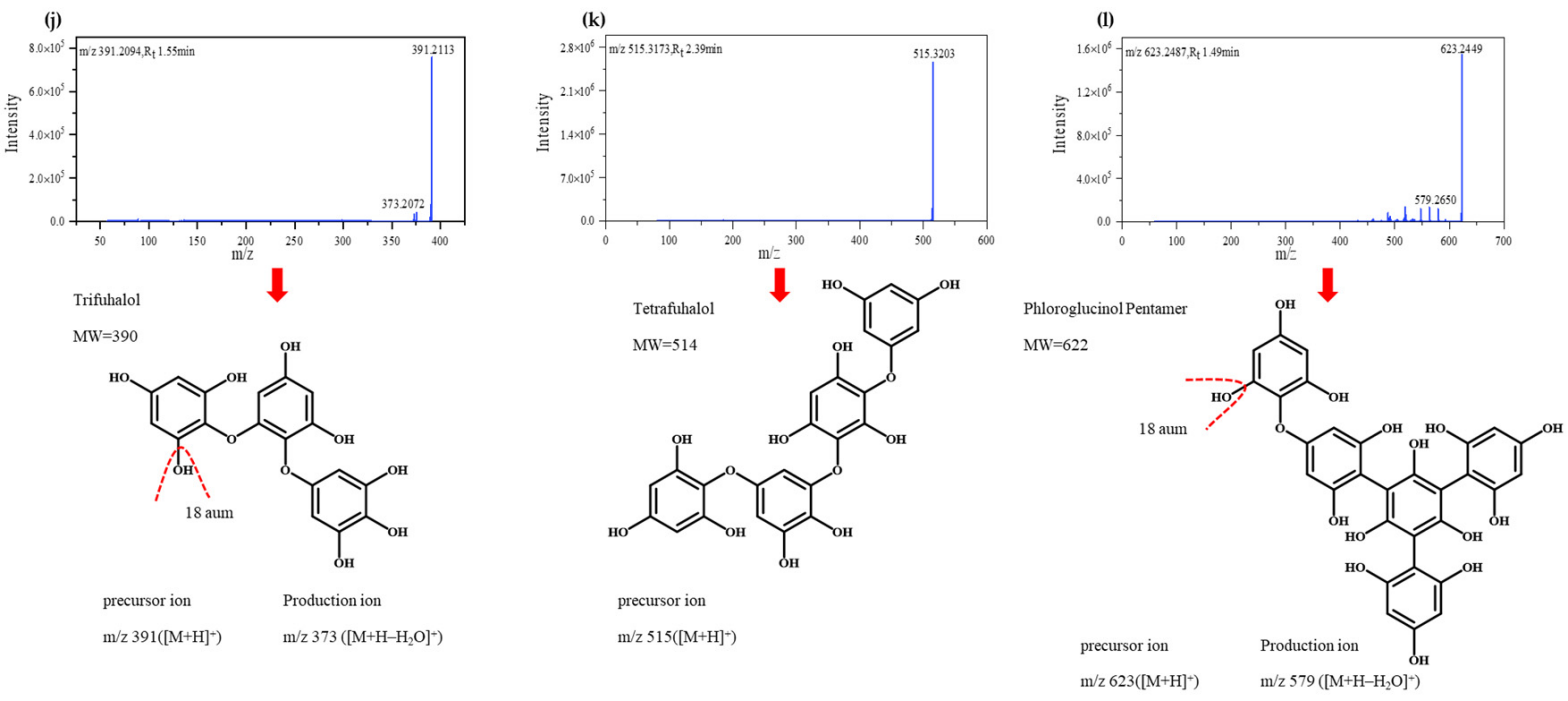
MS/MS fragmentation pattern of identified polyphenolic compounds in Solid-Phase Extraction (SPE) polyphenolic fraction of brown macroalgae. Polyphenolic compounds’ molecular structures were obtained from http://www.chemspider.com/ database.

Q-Exactive HF-X mass spectrometry was used to identify polyphenolic compounds in the evaluated brown macroalgae. Twelve compounds were detected using this method which belonged to various classes: phenolic acids (*n* = 5), flavonoid (*n* = 1) and phlorotannins (*n* = 6). Phenolic acids were 4-hydroxybenzoic acid, vanillic acid, gallic acid, caffeic acid, and ferulic acid. The identified flavonoid was epicatechin. Six special classes of polyphenolics were detected. They were phloroglucinol, bifuhalol, phloroglucinol trimer, trifuhalol, tetrafuhalol, and phloroglucinol pentamer. Detected polyphenolics were further identified by comparison of obtained mass spectra with previously available data. Retention times (Rt), precursor ions, fragment ions, and structural formula for each identified polyphenolic compound are presented in Table 1. Q-Exactive HF-X secondary mass spectra of identified peaks are provided in Figure 2 along with the structural formula and mass spectral fragmentation of identified polyphenolic compounds. 

Q-Exactive HF-X spectrum of compound 1 showed an absorption peak at Rt = 6.16 min, and its parent ion was detected at m/z 127.0865, being thus identified as phloroglucinol; a fragment ion was observed at m/z 109.0736 ([M+H]^+^-18amu), which might be caused by the loss of one molecule of water, and the peak was further confirmed by comparing its fragmentation pattern with previously reported data [24]. Phloroglucinol was previously reported that it showed antioxidant activity in brown macroalgae by Kang et al. [36]. Compound 2 was identified as 4-hydroxybenzoic acid [32] and compound 3 as vanillic acid [37], and mass spectra of both compounds correspondingly showed the loss of one molecule of water. The absorption peak of compound 4 was detected at Rt = 6.30 min and fragment ions were detected at m/z 153.065 and m/z 127.0703, which may be due to the loss of one molecule of water ([M+H]^+^-18 aum) or carboxylic acid ([M+H]^+^-44aum) [38]. Compound 5 (m/z 181.0580; Rt = 8.24 min) was identified as caffeic acid based on previous research [37]; fragment ions at m/z 137.0461 and m/z 119.0582 were observed, the latter likely being generated by the combined elimination of an ethylene group and a molecule of water as a consequence of the internal cleavage of benzene ring structures [39]. Compound 6 was refereed to be ferulic acid [40], and significant losses of 18 (H_2_O) and 44 (COOH) amu were detected. Compared with previous research, compound 7 (m/z 267.0949; Rt = 3.37 min) was identified as bifuhalol, a type of phlorotannin [41], and its fragmentation patterns were similar to those of compound 5. Compound 8 was identified as a type of flavonoid, namely epicatechin [32], being detected at m/z 291.0691, whose fragmentation rule was deduced in Mass Frontier 7.0 (Figure 2h); fragment ions were detected at m/z 157.028. The remainder compounds (9–12) were identified as phlorotannins. Compound 9 was viewed as a phloroglucinol trimer, which is a common phlorotannin occurring in several seaweed species of the orders Fucales and Laminariales [24,39]. According to results obtained by Zhang et al. [41], compound 10 (m/z 391.2094; Rt = 1.55 min) was identified as trifuhalol and compound 11 as tetrafuhalol. Compounds 9 and 10 lost one molecule of water ([M+H]^+^-18 aum), while compound 11 (m/z 515.3170; Rt = 2.65 min) did not yield any fragment ions, which has been observed previously [41]. Finally, compound 12 (m/z 623.2487; Rt = 1.49 min) was viewed as phloroglucinol pentamers [42] showing the loss of one molecule of water ([M+H]^+^-18 aum).

### 3.3. Metabolomic Tools for Profiling Polyphenolic Compounds

Multivariate data analysis has been increasingly regarded as an important tool in the food industry. In particular, PCA has been used to differentiate and classify food products according to geographical origin, as well as to implement a chemotaxonomic approach to botanical classification. In the present study, multivariate statistical analyses were used to determine the distinct composition and behavioral patterns of polyphenolic compounds in four brown macroalgae species. 

**Figure 3:**
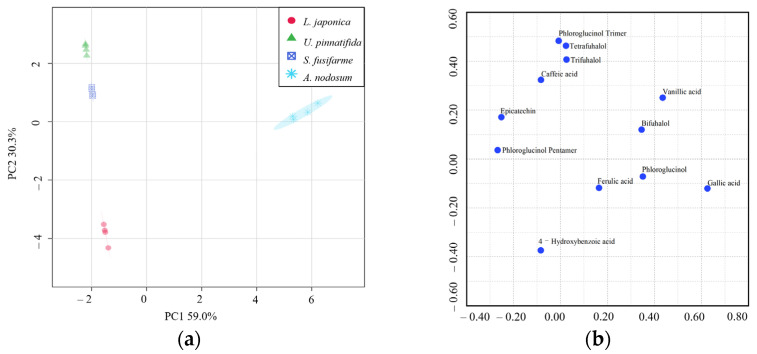
Principal component analysis (PCA) score plot (**a**) and PCA loadings plot (**b**) for four types of brown macroalgae.

PCA was conducted to evaluate variation in the dataset when reduced and redistributed linearly and also randomly in scores (species) and loadings (polyphenolic compounds). Figure 3a depicts the PCA score plot of components 1 and 2 which explain 89.3% of the total variance. PCA plot showed clear differentiation in polyphenolic profiles among evaluated brown macroalgae species. Interspecific variation in low molecular weight phlorotannin profiles in certain brown macroalgae species has been previously reported. For instance, phlorotannins profiles in *Pelvetia canaliculata*, *Fucus vesiculosus*, *A. nodosum,* and *Himanthalia elongata* were shown to differ according to the species and the season [30]. Figure 3b depicts the PCA loading plot with the distribution of polyphenolic profiles of all four brown macroalgae. The PCA score plot and the PCA loading plot showed great correspondence in spatial position. Polyphenolic compounds in *A. nodosum* were mainly vanillic acid, gallic acid, ferulic acid, phloroglucinol, and bifuhalol. 4-hydroxybenzoic acid was the main polyphenolic compound in *L. japonica*.

**Figure 4:**
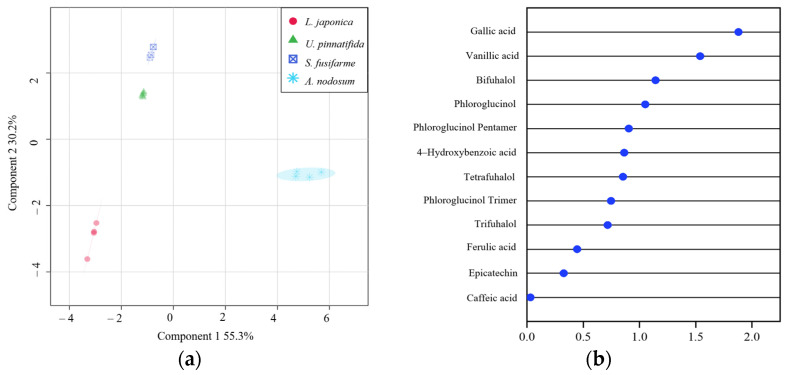
Partial least squares discriminant analysis (PLS-DA) score plot (**a**) and VIP scores by PLS-DA (**b**) derived from Q-Exactive HF-X mass spectrometry data using positive electrospray ionization of polyphenolic compounds in four types of brown macroalgae.

To obtain a more distinct discrimination of samples and determine which metabolites contribute to such discrimination, PLS-DA was performed on obtained Q-Exactive HF-X mass spectrometric data. For the PLS-DA model, values of Q^2^ max (0.76) and R^2^ (0.83) were higher in the permutation test than in the real model, suggesting good predictability and goodness of fit. Figure 4a provides relevant information for species separation as identified by PLS-DA.

According to the PCA and PLS-DA score map, A. nodosum was separated from *L. japonica*, *S. fusiforme,* and *U. pinnatifida* in the first principal component (PC 1). Moreover, *A. nodosum* and *L. japonica* were separated from *S. fusiforme* and *U. pinnatifida* in PC 2. VIP score is a weighted sum of squares of the PLS-DA loadings and were used to determine which polyphenolic compounds contributed to the separation into the aforementioned groups (Figure 4b). Polyphenolic compounds that presented VIP scores greater than 0.9 and likely have contributed to discriminating polyphenolic profiles in evaluated macroalgae were gallic acid (VIP score = 1.88), vanillic acid (VIP score = 1.54), bifuhalol (VIP score = 1.14), phloroglucinqol (VIP score = 1.05) and phloroglucinol pentamer (VIP score = 0.91).

### 3.4. Correlation between Polyphenolic Compounds and Antioxidant Capacity

**Figure 5:**
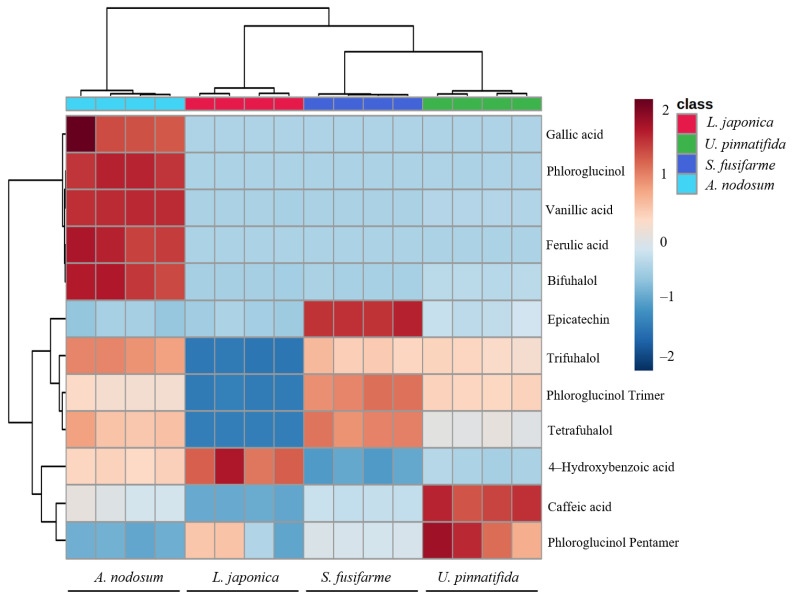
Hierarchical cluster analysis (HCA) and heatmap of polyphenolic compounds in four types of brown macroalgae.

Through q-Exactive HF-X analysis data, a relative quantitative heat map (Figure 5) was drawn to identify the variation pattern of polyphenolics content in four evaluated brown macroalgae. Based on results of DPPH free radical scavenging and H_2_O_2_-challenged cell viability assays, the antioxidant activity of brown macroalgae extracts was highest in *A. nodosum* and lowest in *L. japonica* (Figure 1a). Total phenolic content in *A. nodosum* was the highest compared to other evaluated brown macroalgae, whose levels of gallic acid, phloroglucinol, vanillic acid, ferulic acid, and bifuhalol were significantly higher, and was shown to contain other polyphenolics such as trifuhalol, phloroglucinol trimer, tetrafuhalol, and 4-hydroxybenzoic acid. Therefore, the *A. nodosum* extract was found to possess the strongest antioxidant activity when confronting all four brown macroalgae extracts. Although total phenolic content in *U. pinnatifida* was higher than that in *S. fusiforme*, the higher antioxidant activity found in *S. fusiforme* extract might be related to a higher content of trifuhalol, phloroglucinol trimer, and tetrafuhalol, and the effect of polyphenolic compounds on antioxidant activity was also reported by Kirke et al. [30]. So the biological activity of polyphenolics in algae has been shown to be significantly affected by the type and structure of polyphenolic compounds. More specifically, simple polyphenolics have one hydroxyl (-OH) group attached to a carbon atom in a benzene ring; catechol and intercal hydroquinone (benzenediol) have two -OH groups; and pyrogallol and phloroglucinol (benzenetriol) have three -OH groups [6]. It has also been demonstrated that the effectiveness and biological activity of polyphenolics depend on the resonance stability of phenoxy radicals, while resonance stability of phenoxy radicals is affected by the number of substituents (relative to -OH groups) in ortho and para position in aromatic rings [22]. Phlorotannins are the most studied group of polyphenolics in marine macroalgae and can have up to eight interconnected rings which are derived from phloroglucinol (1,3,5-trihydroxybenzene) monomer units [11]. Therefore, phlorotannins are a more potent scavenger of free radicals than other polyphenols derived from terrestrial plants, including green tea catechins [3]. Finally, as seen in Figure 5, the varieties and content of polyphenols in *L. japonica* were low, which also contributed to its weaker antioxidant activity compared to other tested brown macroalgae.

## 4. Conclusions

Differences in polyphenolic compounds and antioxidant activity of four brown macroalgae species were determined. Q-Exactive HF-X mass spectrometry was used to identify and characterize polyphenolic compounds. A total of 12 polyphenolic compounds were identified, including 5 phenolic acids, 1 flavonoid, and 6 phlorotannins. Through multivariate analysis (PCA and PLS-DA), polyphenolic compounds and antioxidant activity were found to significantly differ among evaluated species of brown macroalgae. Total phenolic content and antioxidant activity were highest in *A. nodosum* and lowest in *L. japonica*. In addition, the antioxidant activity of polyphenolics in all four brown macroalgae was shown to be affected by polyphenolic compounds, especially phlorotannins. Interestingly, phlorotannins were uniquely found in brown algae and showed great antioxidant properties and other physiological activities. Collectively, *A. nodosum* was shown to be a good source of natural polyphenolics with strong antioxidant activity. This highlights that this species is an ideal marine polyphenolics enrichment species for future commercial applications, as well as its applicability as a model species for further study of phlorotannins.

## Figures and Tables

**Table 1:** Identification of bioactive compounds in the polyphenolic fraction of Solid-Phase Extraction (SPE) obtained from *L. japonica*, *U. pinnatifida*, *S. fusiforme,* and *A. nodosum* using Q-Exactive™ HF-X Hybrid Quadrupole-Orbitrap™ mass spectrometry analysis.

Compound	Rt(min)	MS^1^[M+H]^+^	Identification	Molecular Formula	References
1	6.16	127.0865	Phloroglucinol	C_6_H_6_O_3_	[23]
2	2.85	139.0498	4-Hydroxybenzoic acid	C_7_H_6_O_3_	[30]
3	1.79	169.0969	Vanillic acid	C_8_H_8_O_4_	[35]
4	6.30	171.0758	Gallic acid	C_7_H_6_O_5_	[36]
5	8.24	181.0580	Caffeic acid	C_9_H_8_O_4_	[35]
6	7.68	195.0737	Ferulic acid	C_10_H_10_O_4_	[38]
7	3.37	267.0949	Bifuhalol	C_12_H_10_O_7_	[39]
8	6.09	291.0691	Epicatechin	C_15_H_14_O_6_	[30]
9	1.41	375.2131	Phloroglucinol Trimer	C_18_H_14_O_9_	[23,37]
10	1.55	391.2094	Trifuhalol	C_18_H_14_O_10_	[39]
11	2.39	515.3173	Tetrafuhalol	C_24_H_18_O_13_	[39]
12	1.49	623.2487	Phloroglucinol Pentamer	C_30_H_22_O_15_	[40]

Rt: retention time in minutes; MS^1^[M+H]^+^: molecular ion.

## Data Availability

The data showed in this study are contained within the article.

## References

[1] Deliza R., Rosenthal A., Silva A.L.S. (2003). Consumer attitude towards information on non conventional technology. Trends Food Sci. Technol..

[2] Plaza M., Cifuentes A., Ibanez E. (2008). In the search of new functional food ingredients from algae. Trends Food Sci. Technol..

[3] Abdelhamid A., Jouini M., Bel Haj Amor H., Mzoughi Z., Dridi M., Ben Said R., Bouraoui A. (2018). Phytochemical analysis and evaluation of the antioxidant, anti-Inflammatory, and antinociceptive potential of phlorotannin-rich fractions from three mediterranean brown seaweeds. Mar. Biotechnol..

[4] FAO (2018). The State of World Fisheries and Aquaculture. http://www.fao.org/3/i9540en/i9540en.pdf.

[5] Dixit D.C., Reddy C.R.K., Balar N., Suthar P., Gajaria T., Gadhavi D.K. (2017). Assessment of the nutritive, biochemical, antioxidant and antibacterial potential of eight tropical macro algae along kachchh coast, india as human food supplements. J. Aquat. Food Prod. Technol..

[6] Generalić Mekinić I., Skroza D., Šimat V., Hamed I., Čagalj M., Popović Perković Z. (2019). Phenolic Content of Brown Algae (*Pheophyceae*) Species: Extraction, Identification, and Quantification. Biomolecules.

[7] Dai J., Mumper R.J. (2010). Plant phenolics: Extraction, analysis and their antioxidant and anticancer properties. Molecules.

[8] Fraga C.G., Galleano M., Verstraeten S.V., Oteiza P.I. (2010). Basic biochemical mechanisms behind the health benefits of polyphenols. Mol. Asp. Med..

[9] Martinez J.H., Castaneda H.G. (2013). Preparation and chromatographic analysis of phlorotannins. J. Chromatogr. Sci..

[10] Dang T.T., Bowyer M.C., Van Altena I.A., Scarlett C.J. (2018). Comparison of chemical profile and antioxidant properties of the brown algae. Int. J. Food Sci. Technol..

[11] Koivikko R., Loponen J., Honkanen T., Jormalainen V. (2005). Contents of soluble, cell-wall-bound and exuded phlorotannins in the brown alga *Fucus vesiculosus*, with implications on their ecological functions. J. Chem. Ecol..

[12] Li Y., Qian Z.J., Ryu B., Lee S.H., Kim M.M., Kim S.K. (2009). Chemical components and its antioxidant properties in vitro: An edible marine brown alga, *Ecklonia cava*. Bioorgan. Med. Chem..

[13] Balboa E.M., Conde E., Moure A., Falque E., Dominguez H. (2013). In vitro antioxidant properties of crude extracts and compounds from brown algae. Food Chem..

[14] Heffernan N., Brunton N.P., FitzGerald R.J., Smyth T.J. (2015). Profiling of the molecular weight and structural isomer abundance of macroalgae-derived phlorotannins. Mar. Drugs.

[15] Wijesinghe W.A., Jeon Y.J. (2012). Exploiting biological activities of brown seaweed *Ecklonia cava* for potential industrial applications: A review. Int. J. Food Sci. Nutr..

[16] Kang M.C., Cha S.H., Wijesinghe W.A., Kang S.M., Lee S.H., Kim E.A., Song C.B., Jeon Y.J. (2013). Protective effect of marine algae phlorotannins against AAPH-induced oxidative stress in zebrafish embryo. Food Chem..

[17] Kim A.R., Shin T.S., Lee M.S., Park J.Y., Park K.E., Yoon N.Y., Kim J.S., Choi J.S., Jang B.C., Byun D.S. (2009). Isolation and identification of phlorotannins from *Ecklonia stolonifera* with antioxidant and anti-inflammatory properties. J. Agric. Food Chem..

[18] Nwosu F., Morris J., Lund V.A., Stewart D., Ross H.A., McDougall G.J. (2011). Anti-proliferative and potential anti-diabetic effects of phenolic-rich extracts from edible marine algae. Food Chem..

[19] Steevensz A.J., Mackinnon S.L., Hankinson R., Craft C., Connan S., Stengel D.B., Melanson J.E. (2012). Profiling phlorotannins in brown macroalgae by liquid chromatography-high resolution mass spectrometry. Phytochem. Anal..

[20] Murray M., Dordevic A.L., Ryan L., Bonham M.P. (2017). An emerging trend in functional foods for the prevention of cardiovascular disease and diabetes: Marine algal polyphenols. Crit. Rev. Food Sci..

[21] Tierney M.S., Soler-Vila A., Rai D.K., Croft A.K., Brunton N.P., Smyth T.J. (2013). UPLC-MS profiling of low molecular weight phlorotannin polymers in *Ascophyllum nodosum*, *Pelvetia canaliculata* and *Fucus spiralis*. Metabolomics.

[22] Hermund D.B., Plaza M., Turner C., Jonsdottir R., Kristinsson H.G., Jacobsen C., Nielsen K.F. (2018). Structure dependent antioxidant capacity of phlorotannins from Icelandic *Fucus vesiculosus* by UHPLC-DAD-ECD-QTOFMS. Food Chem..

[23] Audibert L., Fauchon M., Blanc N., Hauchard D., Gall E.A. (2010). Phenolic compounds in the brown seaweed *Ascophyllum nodosum*: Distribution and radical-scavenging activities. Phytochem. Anal..

[24] Ferreres F., Lopes G., Gil-Izquierdo A., Andrade P.B., Sousa C., Mouga T., Valentao P. (2012). Phlorotannin extracts from fucales characterized by HPLC-DAD-ESI-MSn: Approaches to hyaluronidase inhibitory capacity and antioxidant properties. Mar. Drugs.

[25] Stengel D.B., Connan S., Popper Z.A. (2011). Algal chemodiversity and bioactivity: Sources of natural variability and implications for commercial application. Biotechnol. Adv..

[26] Ummat V., Tiwari B.K., Jaiswal A.K., Condon K., Garcia-Vaquero M., O’Doherty J., O’Donnell C., Rajauria G. (2020). Optimisation of Ultrasound Frequency, Extraction Time and Solvent for the Recovery of Polyphenols, Phlorotannins and Associated Antioxidant Activity from Brown Seaweeds. Mar. Drugs.

[27] Riitta J.T. (1985). Phenolic constituents in the leaves of northern willows methods for the analysis of certain phenolics. J. Agric. Food Chem..

[28] Kawasaki K., Yin J.J., Subczynski W.K., Hyde J.S., Kusumi A. (2001). Pulse EPR Detection of Lipid Exchange between Protein-Rich Raft and Bulk Domains in the Membrane: Methodology Development and Its Application to Studies of Influenza Viral Membrane. Biophys. J..

[29] Qi H., Dong X.F., Zhao Y.P., Li N., Fu H., Feng D.D., Liu L., Yu C.X. (2016). ROS production in homogenate from the body wall of sea cucumber *Stichopus japonicus* under UVA irradiation: ESR spin-trapping study. Food Chem..

[30] Kirke D.A., Rai D.K., Smyth T.J., Stengel D.B. (2019). An assessment of temporal variation in the low molecular weight phlorotannin profiles in four intertidal brown macroalgae. Algal Res..

[31] Kuda T., Kunii T., Goto H., Suzuki T., Yano T. (2007). Varieties of antioxidant and antibacterial properties of *Ecklonia stolonifera* and *Ecklonia kurome* products harvested and processed in the Noto peninsula, Japan. Food Chem..

[32] Machu L., Misurcova L., Vavra Ambrozova J., Orsavova J., Mlcek J., Sochor J., Jurikova T. (2015). Phenolic content and antioxidant capacity in algal food products. Molecules.

[33] Otero P., López-Martínez M.I., García-Risco M.R. (2019). Application of pressurized liquid extraction (PLE) to obtain bioactive fatty acids and phenols from *Laminaria ochroleuca* collected in Galicia (NW Spain). J. Pharm. Biomed. Anal..

[34] Kang K.A., Lee K.H., Chae S., Zhang R., Jung M.S., Lee Y., Kim S.Y., Kim H.S., Joo H.G., Park J.W. (2005). Eckol isolated from *Ecklonia cava* attenuates oxidative stress induced cell damage in lung fibroblast cells. FEBS Lett..

[35] Barros Santos M.C., Ribeiro da Silva Lima L., Ramos Nascimento F., Pimenta do Nascimento T., Cameron L.C., Simoes Larraz Ferreira M. (2019). Metabolomic approach for characterization of phenolic compounds in different wheat genotypes during grain development. Food Res. Int..

[36] Kang K.A., Zhang R., Chae S., Lee S.J., Kim J., Kim J., Jeong J., Lee J., Shin T., Lee N.H. (2010). Phloroglucinol (1,3,5-trihydroxybenzene) protects against ionizing radiation-induced cell damage through inhibition of oxidative stress in vitro and in vivo. Chem-Biol. Interact..

[37] Klejdus B., Plaza M., Snoblova M., Lojkova L. (2017). Development of new efficient method for isolation of phenolics from sea algae prior to their rapid resolution liquid chromatographic-tandem mass spectrometric determination. J. Pharm. Biomed. Anal..

[38] Rajauria G., Foley B., Abu-Ghannam N. (2016). Identification and characterization of phenolic antioxidant compounds from brown Irish seaweed *Himanthalia elongata* using LC-DAD–ESI-MS/MS. Innov. Food Sci. Emerg..

[39] Wang T., Jonsdottir R., Liu H., Gu L., Kristinsson H.G., Raghavan S., Olafsdottir G. (2012). Antioxidant capacities of phlorotannins extracted from the brown algae *Fucus vesiculosus*. J. Agric. Food Chem..

[40] Rajauria G. (2018). Optimization and validation of reverse phase HPLC method for qualitative and quantitative assessment of polyphenols in seaweed. J. Pharm. Biomed. Anal..

[41] Zhang R., Yuen A.K.L., Magnusson M., Wright J.T., Nys R., Masters A.F., Maschmeyer T. (2018). A comparative assessment of the activity and structure of phlorotannins from the brown seaweed *Carpophyllum flexuosum*. Algal Res..

[42] Lopes G., Barbosa M., Vallejo F., Gil-Izquierdo Á., Andrade P.B., Valentão P., Pereira D.M., Ferreres F. (2018). Profiling phlorotannins from *Fucus* spp. of the Northern Portuguese coastline: Chemical approach by HPLC-DAD-ESI/MS and UPLC-ESI-QTOF/MS. Algal Res..

